# Same, but different: Binding effects in auditory, but not visual detection performance

**DOI:** 10.3758/s13414-021-02436-5

**Published:** 2022-02-02

**Authors:** Lars-Michael Schöpper, Christian Frings

**Affiliations:** Department of Cognitive Psychology, University of Trier, Trier, Germany

**Keywords:** S-R binding, attention, perception, target modality

## Abstract

Responding to a stimulus leads to the integration of response and stimulus’ features into an event file. Upon repetition of any of its features, the previous event file is retrieved, thereby affecting ongoing performance. Such integration-retrieval explanations exist for a number of sequential tasks (that measure these processes as ’binding effects’) and are thought to underlie all actions. However, based on attentional orienting literature, Schöpper, Hilchey, et al. (2020) could show that binding effects are absent when participants detect visual targets in a sequence: In visual detection performance, there is simply a benefit for target location changes (inhibition of return). In contrast, Mondor and Leboe (2008) had participants detect auditory targets in a sequence, and found a benefit for frequency repetition – presumably reflecting a binding effect in auditory detection performance. In the current study, we conducted two experiments, that only differed in the modality of the target: Participants signaled the detection of a sound (N = 40) or of a visual target (N = 40). Whereas visual detection performance showed a pattern incongruent with binding assumptions, auditory detection performance revealed a non-spatial feature repetition benefit, suggesting that frequency was bound to the response. Cumulative reaction time distributions indicated that the absence of a binding effect in visual detection performance was not caused by overall faster responding. The current results show a clear limitation to binding accounts in action control: Binding effects are not only limited by task demands, but can entirely depend on target modality.

## Introduction

If our phone lights up, we unlock it to see if someone wrote a message. When it rings, we press a button to take the call. Both of these movements are defined as actions, because they have an intention and anticipated goal in mind (e.g., Frings et al., 2020; Prinz, 1998), and thus are in the scope of action control theories. According to such theories, when responding to a stimulus (like a ringing phone), the stimulus’ features, even if task-irrelevant (Frings et al., 2007), and the response are integrated into a short episodic memory trace – an event file (e.g., Frings et al., 2020; Hommel, 1998, 2004; Hommel et al., 2001). Repeating any of its components, the previous event file gets retrieved, typically leading to benefits for full repetitions, but interference for partial repetitions. The resulting so-called *stimulus-response (S-R) binding effects* can be measured in prime-probe sequences, in which participants respond to a first target in a prime display, followed by a response to a second target in a probe display (e.g., Frings et al., 2007; Singh et al., 2016). From prime to probe, a response-defining feature and a response-irrelevant feature is orthogonally varied to repeat or change. Crucially, when information from prime to probe only partially repeats (e.g., response repetition with change of response-irrelevant feature), probe reaction times and/or error rates increase (e.g., Frings et al., 2007; Hilchey et al., 2018; Schöpper, Singh, & Frings, 2020; Singh et al., 2016).

**Fig. 1:**
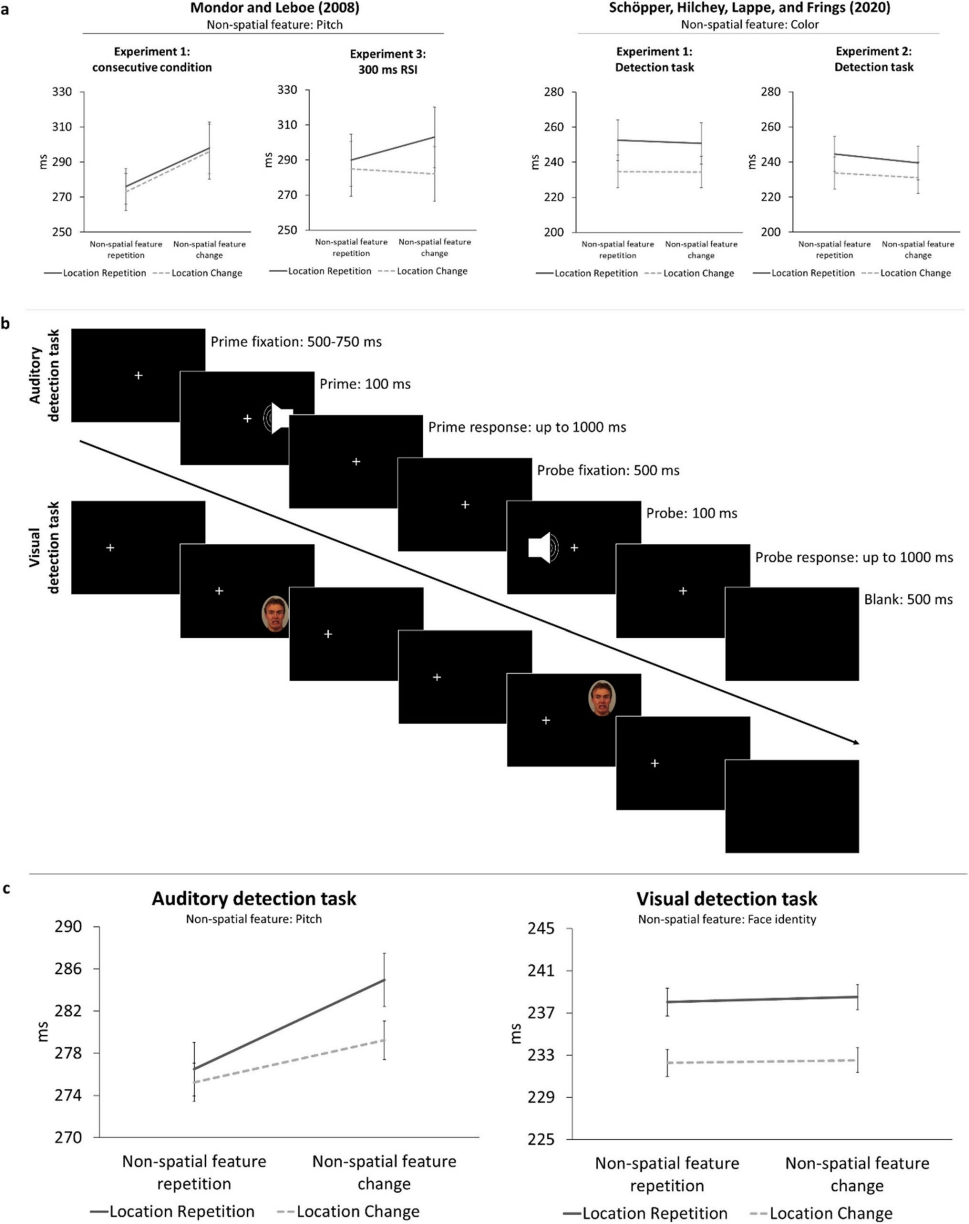
**a**) Left column: Data redrawn for the experiments of interest from Mondor and Leboe (2008); error bars represent standard error as reported in the paper. Right column: Data redrawn for the detection tasks from Schöpper, Hilchey, et al. (2020); error bars represent standard error of the mean. **b**) Trial sequence as used in the auditory (top row) and visual (bottom row) detection task. The above trial sequences depict trials in which the non-spatial feature repeats – in the auditory detection task the same frequency represented by a placeholder symbol, in the visual detection task the same face (picture ID “AM08AFS” in Lundqvist et al., 1998) – whereas the location changes (i.e., in both cases a FRLC-trial, see main text). c) Reaction times in ms for the auditory detection task (left panel) and visual detection task (right panel). Error bars represent within standard error for each task following Cousineau (2005) with correction by Morey (2008)

Although top-down (e.g., intentional weighting; Memelink & Hommel, 2013) and bottom-up factors (e.g., figure-ground segmentation; Frings & Rothermund, 2017) have been found to modulate the processes leading to binding effects (see Frings et al., 2020), these processes are implicitly assumed to underlie all actions (e.g., Frings et al., 2020; Hommel, 2004; Prinz, 1998). Yet, the attentional orienting literature (Hilchey et al., 2018; Huffman et al., 2018) suggests an absence of feature integration effects in tasks where participants signal the detection or location of visual stimuli. In these tasks, a benefit for location changes, that is, *inhibition of return* (IOR; Klein, 2000), is observed. Spurred on by this, Schöpper, Hilchey, et al. (2020) investigated if binding effects are absent in simple visual detection performance. According to Schöpper, Hilchey, et al. (2020; see also Huffman et al., 2018), binding in detection performance in a task with color and location of stimuli as irrelevant features could manifest in three possible outcomes. An interaction of color and location could be caused by binding of the location feature and color feature (i.e., binding between features irrespective of the response; Hommel, 2004; Kahneman et al., 1992; Treisman & Gelade, 1980), which would manifest in an X-shaped pattern: Benefits for full repetition and full change of location and color, and interference for partial repetitions (Hommel, 2004). A main effect of color or a main effect of location, caused by binding of color and response or location and response, would manifest in a benefit for the repetition of the color or location feature, respectively: If the same detection response is given to every stimulus, all responses are response repetitions for which the repetition of a (task-irrelevant) feature should be beneficial – at least in terms of binding and retrieval. In two experiments, participants signaled the detection of a dot repeating or changing its color, as well as repeating or changing its location. In stark contrast to binding assumptions in action control theories, but fully in accordance with the attentional orienting literature (Huffman et al., 2018), visual detection performance yielded IOR without any modulation by color repetition or change (see Fig. 1a, right column). It was argued that binding effects in detection performance are absent – and thus incongruent with the assumed ubiquity of binding effects underlying all actions – because non-spatial target features do not have to be processed for responding (Huffman et al., 2018; Schöpper, Hilchey, et al., 2020).

Yet, a study by Mondor and Leboe (2008) challenges this ‘boundary of binding theories’. Here, participants signaled the detection of a sound repeating or changing its frequency while repeating or changing the ear to which it was played. When participants gave a detection response^1^ to every frequency (Experiment 1: Consecutive condition), and when the response-stimulus interval (RSI) between subsequent targets was short (Experiment 3: 300 ms RSI condition), there was a benefit if frequency repeated (see Fig. 1a, left column). In line with the interpretation by Mondor and Leboe (2008), such a main effect resembles binding between non-spatial feature and response, a prediction proposed but falsified for visual detection performance by Schöpper, Hilchey, et al. (2020): If a participant detects a non-spatial feature by pressing a button, the information is integrated. Because in detection performance every response execution resembles a response repetition (i.e., the same response is given to all stimuli), a feature repetition should be beneficial, as the event file can be fully retrieved – while a feature change should slow down responding.

The question then emerges, why processing of visual and auditory information is different when performing the exact same (detection) tasks in which non-spatial features are irrelevant for response generation. A modality-specific view would be supported by specific (neurophysiological) processing of visual and auditory information, with the most obvious being the differentiation into a visual and auditory cortex (e.g., Bear et al., 2007). A modality-invariant explanation might argue that auditory information can be alerting (van der Lubbe & Postma, 2005), distracting (e.g., Escera et al., 1998), and hard to ignore (Spence, Ranson, & Driver, 2000). Accordingly, auditory information might receive so much automatic attention allocation that non-spatial target identity processing becomes inevitable. In fact, attention can have a strong modulating role on the occurrence of binding effects (e.g., Hommel et al., 2014; Moeller & Frings, 2014; Singh et al., 2018).

Thus, it should be possible to observe binding effects in visual detection performance, if the non-spatial information of visual stimuli is harder to ignore. Faces are visual stimuli that typically allocate more attention (see also Theeuwes & Van der Stigchel, 2006) than simple color dots or shapes (see, e.g., Palermo & Rhodes, 2007; Ro et al., 2001), are stimuli that humans perceive very fast (e.g., Ghuman et al., 2014), and have been used to induce IOR in cue-target designs (e.g., Taylor & Therrien, 2005, 2008). Moreover, humans attend to threatful faces (Mogg & Bradley, 1999), and disengage slower from such fearful faces (e.g., Georgiou et al., 2005). Thus, (emotional) faces might be a way better candidate than low-level features as color or shape when comparing visual and auditory targets regarding the allocation of attention they receive. In addition, for retrieval to affect current performance response times are quite crucial; that is, if responding is so fast that retrieval cannot affect upon responding, no binding effect will be observed (see General Discussion). Faces are quite complex visual stimuli when compared with, for example, simple shapes and colors (c.f., Moeller et al., 2016); hence, more complex visual stimuli like faces might help making the response times more comparable between vision and audition.

In the current study, we conducted two separate detection tasks that used a slightly modified version of the visual detection task of Schöpper, Hilchey, et al. (2020). Whereas the auditory detection task used two different frequencies, the visual detection task used two different fearful faces. This allows us to conceptually replicate Mondor and Leboe (2008), and Schöpper, Hilchey, et al. (2020), and to directly compare target modality in a between-experiment comparison with a highly similar design.

## Experiment 1 (Auditory targets)

### Methods

#### Participants

We conducted the auditory (Experiment 1) and visual (Experiment 2) detection task with forty participants, each. This sample size yields a power of 1-β = 0.93 for observing a binding effect with an effect size of *d* = 0.5 (error probability: α = 0.05, one-tailed) in each experiment, and a power of 1-β = 0.60 for observing a modulating role (assumed effect size: *d* = 0.5) of target modality on binding in a between experiment comparison (α = 0.05, two-tailed) (G*Power, Version 3.1.9.2; Faul et al., 2007). Accordingly, forty students (34 women, 6 men, *M*_*age*_ = 22.53, *SD*_*age*_ = 4.03, age range: 18-37 years) of the University of Trier participated in Experiment 1 for either course credit or a monetary reward (5 €), and gave written informed consent. Two participants reported some hearing impairments, but their data was inconspicuous when compared with the sample. None of the other participants reported any hearing impairments.

#### Apparatus and materials

Participants sat in front of a black screen with a white fixation cross at center. Target stimuli were sine wave tones with a frequency of 361 Hz (62 dB; directly measured at earpad) and 712 Hz (74 dB) (frequencies used by Mondor & Leboe, 2008), created in Audacity (Audacity Team), and were presented via headset (Creative Labs Fatal1ty HS-800 Gaming Headset). In contrast to Mondor and Leboe (2008), both frequencies were also slightly distinct by loudness (e.g., as in Schöpper, Singh, & Frings, 2020). Each sound lasted 100 ms, including a linear amplitude on- and offset of 20 ms to avoid on-/ offset clicks. Sounds were programmed to appear 90 % on one headphone channel and 10 % on the other headphone channel to reduce onset clicks in the latter. However, we will refer to the sounds as appearing on the left or right side.

#### Design

The experiment used a 2 (non-spatial feature relation/ pitch: repetition vs. change) x 2 (location relation: repetition vs. change) within-subject design.

#### Procedure

Before instructions were presented on screen, all sounds were played by the program to buffer them. A trial started with a white fixation cross at screen center, remaining until probe response, and participants were instructed to fixate it during a whole trial. After the fixation display (variable duration: 500-750 ms), a sound, that is, the prime target, was played in the left or right headphone channel for 100 ms. Participants signaled the detection of the target by pressing the space bar (with the right index finger) with target onset and up to 1,000 ms after target offset. Missed responses (i.e., no response given 1,000 ms after target offset) were counted as incorrect and produced an error feedback on screen (1,500 ms). After the prime response, there was an interval of 500 ms, followed by the probe target (100 ms) and a detection response was made as described for the prime display. After the probe response, a blank-screen (500 ms) ended a trial and, by that, one prime-probe sequence (see Fig. 1b, top row). Additionally, we included catch trials with no targets throughout a whole prime-probe sequence, that is, trials in which a target was absent in both prime and probe display^2^; here, participants had to wait (1,100 ms for each catch display).

From prime to probe the frequency could repeat (non-spatial feature repetition, FR) or change (non-spatial feature change, FC), and the position of the frequency could repeat (location repetition, LR) or change (location change, LC). Both were orthogonally varied, resulting in four conditions (FRLR, FRLC, FCLR, FCLC). After 19 practice trials with feedback after each prime and probe display, 285 experimental trials followed, comprised of 60 trials for each condition and 45 catch trials. Conditions were drawn randomly; frequencies and positions were pseudo-randomly balanced. After the 95^th^ and 190^th^ trial, participants could take self-paced breaks.

To validate that sounds were localizable to one side, we included a manipulation check at the end of the experiment. Each sound was played three times in random order, resulting in twelve sound presentations to each of which participants gave a left or right keypress response (on arrow keys) without feedback or time pressure.

### Results

#### Manipulation check: Localization of frequencies

Sounds were localized to the side on which they were predominantly programmed in 95.83 % of presentations.

#### Reaction times

For reaction time analysis, we only looked at probe reaction times, as for these repetitions and changes of non-spatial feature and location were systematically varied. Catch trials were excluded from analysis^3^. Only probe reaction times above 50 ms or below 1.5 interquartile range above the third quartile of a participant’s distribution (Tukey, 1977) were included in the analysis. Reaction times were only included if prime and probe responses were correct (i.e., no missed responses). In turn, 5.60 % of trials were discarded.

A 2 (non-spatial feature-relation/ pitch: repetition vs. change) x 2 (location relation: repetition vs. change) repeated-measures ANOVA on probe reaction times yielded a main effect of non-spatial feature relation, *F*(1, 39) = 5.39, *p* = .026, ${\upeta}_p^2$ = .12, with a benefit for frequency repetition (276 ms) over change (282 ms). There was a main effect of location relation, *F*(1, 39) = 7.67, *p* = .009, ${\upeta}_p^2$ = .16, with slower responses for location repetitions (281 ms) compared to changes (277 ms). The interaction of non-spatial feature relation x location relation was not significant, *F*(1, 39) = 2.32, *p* = .136, ${\upeta}_p^2$ = .06 (FRLR: 276 ms; FRLC: 275 ms; FCLR: 285 ms; FCLC: 279 ms; see Fig. 1c, left panel).

#### Distributional analysis of reaction times

It has previously been suggested that retrieval takes time to affect performance (Frings & Moeller, 2012). Accordingly, it has been suggested that detection responses occur so fast that retrieval has no chance to affect performance (Schöpper, Hilchey, et al., 2020; see General Discussion). To test for this, we calculated cumulative reaction time distributions (as in, e.g., Taylor & Ivanoff, 2005). After applying the cut-off criteria mentioned above, we took the 10^th^, 25^th^, 50^th^, 75^th^ and 90^th^ percentile of probe responses for each condition and for each participant (see Table in Appendix). We then conducted a 5 (percentile: 10^th^ vs. 25^th^ vs. 50^th^ vs. 75^th^ vs. 90^th^) x 2 (non-spatial feature relation/ pitch: repetition vs. change) x 2 (location relation: repetition vs. change) repeated-measures MANOVA on probe reaction times. In the auditory detection task, there was a main effect of non-spatial feature relation, *F*(1, 39) = 6.36, *p* = .016, ${\upeta}_p^2$ = .14 (FR: 276 ms; FC: 283 ms), a main effect of location relation, *F*(1, 39) = 6.29, *p* = .016, ${\upeta}_p^2$ = .14 (LR: 282; LC: 278), and a main effect of percentile, *F*(4, 36) = 82.53, *p* < .001, ${\upeta}_p^2$ = .90 (10^th^: 198 ms; 25^th^: 236 ms; 50^th^: 275 ms; 75^th^: 321 ms; 90^th^: 369 ms). The interaction of non-spatial feature relation and percentile was significant, *F*(4, 36) = 3.34, *p* = .020, ${\upeta}_p^2$ = .27. The interaction of location relation and percentile approached, but did not reach significance, *F*(4, 36) = 2.41, *p* = .067, ${\upeta}_p^2$ = .21. No other interaction turned significant (all *F* ≤ 1.62).

**Fig. 2:**
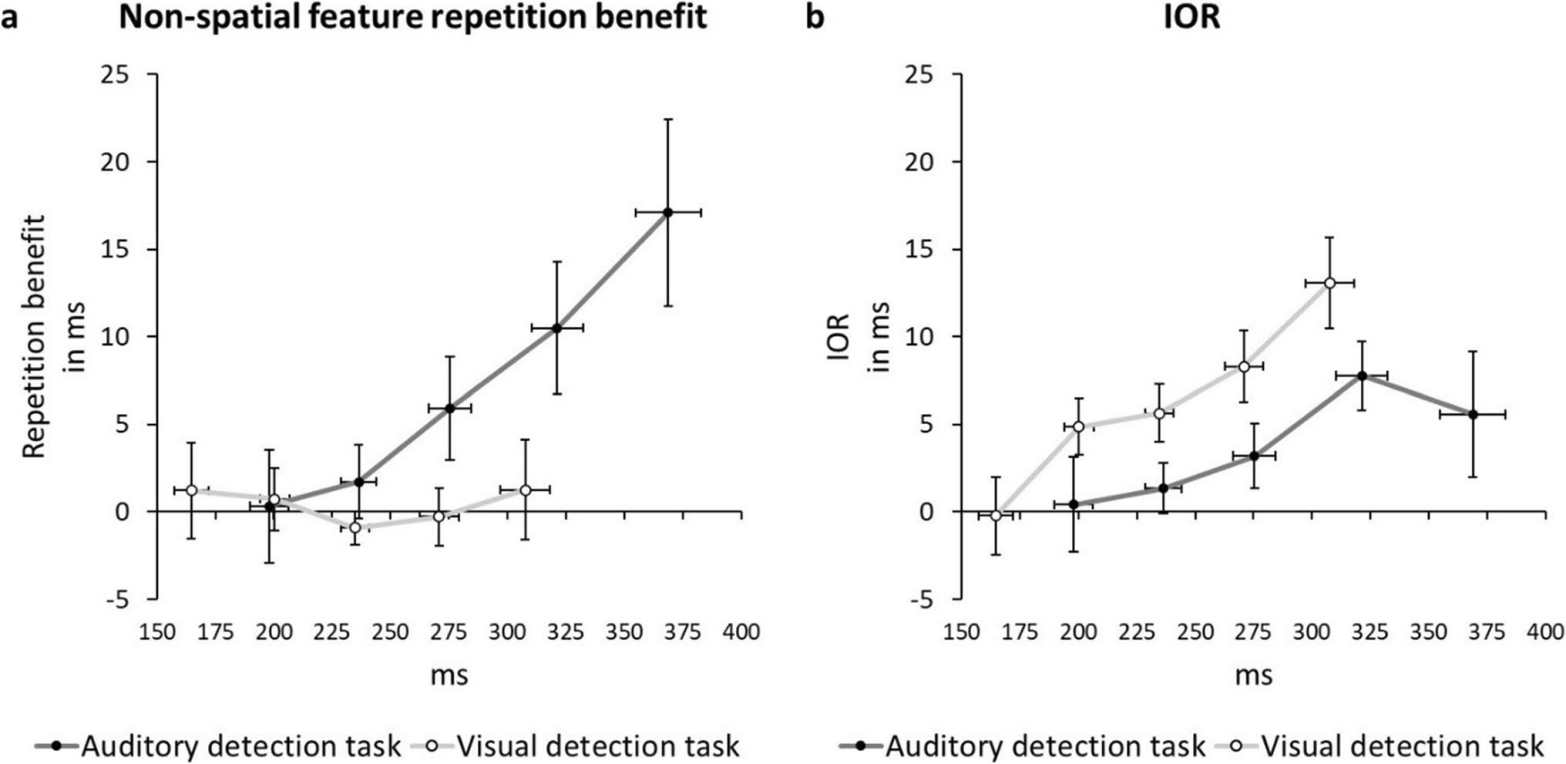
**a**) The calculated non-spatial feature repetition benefit and **b**) the calculated IOR-effect on the y-axis in ms and reaction times on the x-axis in ms as a function of percentile (c.f., delta plots; De Jong et al., 1994; Ridderinkhof, 2002) and experiment. See main text for explanations. The black (auditory detection task) and white (visual detection task) dots represent the 10^th^, 25^th^, 50^th^, 75^th^, and 90^th^ percentile for each function. Error bars represent standard error of each mean of each averaged percentile for the effect of interest (y-axis) and overall reaction time (x-axis)

To highlight the interaction of non-spatial feature relation and percentile, we calculated the main effect of non-spatial feature relation as ((FCLR+FCLC)/2)-((FRLR+FRLC)/2) separate for each percentile, and performed a MANOVA with percentile (10^th^ vs. 25^th^ vs. 50^th^ vs. 75^th^ vs. 90^th^) as a single factor on the calculated non-spatial feature repetition benefit. The main effect of percentile was significant, *F*(4, 36) = 3.34, *p* = .020, ${\upeta}_p^2$ = .27: The non-spatial feature repetition benefit was increasingly pronounced with increasing percentiles (10^th^: 0 ms; 25^th^: 2 ms; 50^th^: 6 ms; 75^th^: 10 ms; 90^th^: 17 ms; see Fig. 2a). For sake of completeness, we calculated the main effect of location relation, that is, IOR, as ((FCLR+FRLR)/2)-((FCLC+FRLC)/2) separate for each percentile. The main effect of percentile approached significance, *F*(4, 36) = 2.41, *p* = .067, ${\upeta}_p^2$ = .21: There was a tendency for IOR to increase with increasing percentiles (10^th^: 0 ms; 25^th^: 1 ms; 50^th^: 3 ms; 75^th^: 8 ms; 90^th^: 6 ms; see Fig. 2b).

### Discussion

In Experiment 1, participants signaled the detection of auditory targets orthogonally repeating or changing their non-spatial feature (i.e., pitch) and location. We replicated Mondor and Leboe (2008) in that we observed a benefit for a frequency repetition. This pattern is congruent with binding between response and non-spatial feature as expected by binding approaches in action control. This effect became larger with increasing response times. Additionally, we found an overall benefit for location changes, that is, IOR.

## Experiment 2 (Visual targets)

### Participants

Forty students (31 women, 9 men, *M*_*age*_ = 22.20, *SD*_*age*_ = 3.38, age range: 18-35 years) of the University of Trier participated for either course credit or a monetary reward (5€), and gave written informed consent. All reported normal or corrected-to-normal vision, and none had participated in Experiment 1.

### Apparatus and materials

Participants sat approximately 60 cm in front of a black screen (display: 1680 x 1050 px) with a white fixation cross (0.38° x 0.38° of visual angle) on the left screen half. Targets were elliptical cutouts (5.15° x 6.77°) of photographs of a male (picture ID: AM08AFS) and female face (picture ID: BF18AFS), both with a fearful expression, from the Karolinska Directed Emotional Faces-Database (Lundqvist et al., 1998). Targets appeared on the right screen half at an upper or lower position^4^, the latter being approximately 8.10° apart (center-to-center). Diagonal distance between fixation cross and a target position was approximately 11.47° (center-to-center).

### Design and Procedure

Experiment 2 was identical to Experiment 1, except for the following. Instead of a sound being played on the left or right headphone channel, a photograph of a face appeared at top or bottom of the right screen half (see Fig. 1b, lower row). There was no manipulation check regarding target locations.

### Results

#### Reaction times

The same cut-off criteria^5^ as in Experiment 1 resulted in 6.69 % of trials being discarded.

The 2 x 2 ANOVA on probe reaction times revealed no main effect of non-spatial feature relation (face identity), *F*(1, 39) = 0.15, *p* = .703, ${\upeta}_p^2$ < .01, but a main effect of location relation, *F*(1, 39) = 21.98, *p* < .001, ${\upeta}_p^2$ = .36: Responses were slower when location repeated (238 ms) compared to changed (232 ms). The interaction of non-spatial feature relation x location relation was not significant, *F*(1, 39) = 0.01, *p* = .919, ${\upeta}_p^2$ = .00 (FRLR: 238 ms; FRLC: 232 ms; FCLR: 238 ms; FCLC: 233 ms; see Fig. 1c, right panel).

#### Distributional analysis of reaction times

As with Experiment 1, we took the 10^th^, 25^th^, 50^th^, 75^th^ and 90^th^ percentile of probe responses for each condition and for each participant (see Table in Appendix). We performed a 5 (percentile) x 2 x 2 MANOVA on probe reaction times. There was no main effect of non-spatial feature relation, *F*(1, 39) = 0.11, *p* = .742, ${\upeta}_p^2$ < .01. There was a main effect of location relation, *F*(1, 39) = 24.94, *p* < .001, ${\upeta}_p^2$ = .39 (LR: 239; LC: 232), and a main effect of percentile, *F*(4, 36) = 111.54, *p* < .001, ${\upeta}_p^2$ = .93 (10^th^: 165 ms; 25^th^: 200 ms; 50^th^: 235 ms; 75^th^: 271 ms; 90^th^: 308 ms). The interaction of non-spatial feature relation and percentile was not significant, *F*(4, 36) = 0.45, *p* = .772, ${\upeta}_p^2$ = .05. The interaction of location relation and percentile was significant, *F*(4, 36) = 3.71, *p* = .013, ${\upeta}_p^2$ = .29. No other interaction turned significant (all *F* ≤ 1.51).

A MANOVA with percentile (10^th^ vs. 25^th^ vs. 50^th^ vs. 75^th^ vs. 90^th^) as a single factor on the calculated non-spatial feature repetition benefit was not significant, *F*(4, 36) = 0.45, *p* = .772, ${\upeta}_p^2$ = .05 (10^th^: 1 ms; 25^th^: 1 ms; 50^th^: -1 ms; 75^th^: 0 ms; 90^th^: 1 ms; see Fig. 2a). The same MANOVA on the calculated IOR-effect revealed a significant main effect of percentile, *F*(4, 36) = 3.71, *p* = .013, ${\upeta}_p^2$ = .29: IOR was increasingly pronounced with increasing percentiles (10^th^: 0 ms; 25^th^: 5 ms; 50^th^: 6 ms; 75^th^: 8 ms; 90^th^: 13 ms; see Fig. 2b).

#### Discussion

In Experiment 2, participants signaled the detection of visual targets orthogonally repeating or changing their non-spatial feature (i.e., face identity) and location. We replicated Schöpper, Hilchey, et al. (2020) in that we observed an overall benefit for location changes, that is, IOR. This effect increased with increasing response times. Neither did we observe a benefit for a non-spatial feature repetition, nor did this effect manifest in slower response times.

### Between experiment comparison

Adding Experiment (auditory vs. visual targets) as a between-subjects^6^ factor to the 2 x 2 repeated measures ANOVA revealed a main effect of target modality, *F*(1, 78) = 13.53, *p* < .001, ${\upeta}_p^2$ = .15, with participants responding faster when detecting visual (235 ms) compared to auditory targets (279 ms). Crucially, the significant main effect of non-spatial feature relation, *F*(1, 78) = 5.39, *p* = .023, ${\upeta}_p^2$ = .07, was modulated by Experiment, *F*(1, 78) = 4.28, *p* = .042, ${\upeta}_p^2$ = .05: There was a benefit for non-spatial feature repetition (276 ms) over change (282 ms) for auditory targets, but not for visual targets (FR: 235 ms; FC: 236 ms).

The main effect of location relation was significant, *F*(1, 78) =27.78, *p* < .001, ${\upeta}_p^2$ = .26, but was not modulated by Experiment, *F*(1, 78) = 1.82, *p* = .181, ${\upeta}_p^2$ = .02. Neither the interaction of non-spatial feature relation x location relation was significant, *F*(1, 78) = 1.69, *p* = .198, ${\upeta}_p^2$ = .02, nor was it modulated by Experiment, *F*(1, 78) = 1.39, *p* = .241, ${\upeta}_p^2$ = .02.

## General Discussion

In two experiments, participants signaled the detection of a target with a key-press. We found a benefit for a target changing its location irrespective of which modality constituted the target (i.e., IOR for visual and auditory stimuli). If the target was auditory, we found a benefit for non-spatial frequency repetition. This can be interpreted as a non-spatial feature being bound to a response and being subsequently retrieved. Crucially, this pattern was completely absent for visual targets. This effect differed significantly between modalities.

Binding approaches in action control implicitly assume that integration and retrieval processes apply to all simple actions, that is, all body movements that are executed with an intention or anticipated goal in mind (e.g., Frings et al., 2020; Prinz, 1998). Thus, signaling the detection of a target classifies as an action (see Schöpper, Hilchey et al., 2020). As has been replicated in the current study, although this does not apply for visual detection performance (Huffman et al., 2018; Schöpper, Hilchey, et al., 2020), auditory detection performance (Mondor & Leboe, 2008) is indeed affected by processes that can be attributed to binding effects. Our results have consequences for those action control theories (see Frings et al., 2020) that assume binding processes underlying all simple actions: Binding effects can be completely dependent on target modality.

In discrimination tasks, visual (e.g., Frings et al., 2007), auditory (e.g., Moeller et al., 2012), and audio-visual (e.g., Schöpper, Singh, & Frings, 2020) stimuli yield strong binding effects, and do not suggest any modality-differences for the underlying binding processes. In sharp contrast, the present results suggest such modality differences for detection performance. Why this modality difference arises still remains somewhat unclear at this moment, but it might be due to different processing of sensory information with a stronger inevitability of auditory information or easier discriminability of auditory information (compared to the visual targets at least as used in the current and previous experiments). It is also possible that the modality difference emerges due to auditory processing being more sensitive to temporal characteristics (e.g., Bizley & Cohen, 2013), having a better temporal resolution compared to visual input (c.f., Shams et al., 2000), or having worse spatial resolution, for example, in distance estimation, compared to visual input (e.g., Loomis et al., 1998; which can be improved with visual cues, Calcagno et al., 2012; Zahorik, 2001). These differences may not only concern the detection of pitch per se but also the integration and retrieval of auditory stimuli with responses. In a similar vein, contrary to visual spatial negative priming (e.g., Christie & Klein, 2001), performance in auditory spatial negative priming does not lead to location inhibition (e.g., Möller et al., 2016), but rather an impairment if the sound at a previous location changes (“feature mismatch”, e.g., Mayr et al., 2009; Möller et al., 2013). Thus, rather automatic retrieval of auditory information (see, e.g., Mayr & Buchner, 2010) might have spurred on the occurrence of binding in auditory detection performance.

The absence of binding effects in detection and localization performance has previously been explained by no need to process non-spatial target identity (e.g., Hilchey et al., 2018; Huffman et al., 2018; Schöpper, Hilchey, et al., 2020) or that a post-selective process (see, e.g., Zehetleitner et al., 2012) after identifying the target is missing, so that partial repetition costs are not observed (Schöpper et al., subm.; see also Hilchey et al., 2020). Here we could replicate Mondor and Leboe (2008) in that signaling the detection of an auditory stimulus results in a pattern congruent with binding assumptions – although non-spatial feature processing was completely irrelevant for responding. Congruently, Dyson (2010) showed that when localizing a sound with repeating or changing task-irrelevant pitch, a data pattern that can be interpreted as depicting partial repetition costs arose. This suggests the occurrence of binding of irrelevant features in auditory detection and localization performance – contrary to an absence in their visual counterparts (e.g., Huffman et al., 2018; Schöpper, Hilchey, et al., 2020).

It has been argued (Schöpper, Hilchey, et al., 2020) that detection performance is so fast, that retrieval processes have no chance to alter it (“horserace-account”, Frings & Moeller, 2012; Neill, 1997). Although detecting auditory targets was slower than detecting visual targets – which could, for example, be attributed to modality-specific processing demands (however, see, e.g., Spence & Driver, 1998, and Spence, Lloyd, et al., 2000, for faster responding to auditory compared to visual stimuli), to vertical vs. horizontal processing (c.f., Snyder & Schmidt, 2014; Soballa et al., in prep.; Spalek & Hammad, 2004), or, given our interpretation, to auditory processing being affected by an additional process (i.e., retrieval) – leaving slightly more time for retrieval, auditory detection performance was still much faster than visual localization performance, in which no binding is observed as well (see Huffman et al., 2018). Yet, it could be that slower visual detection responses are indeed affected by retrieval, but that this is blurred by the majority of fast responses. To test for this, we calculated the cumulative reaction time distributions for each experiment. Although the binding pattern in the auditory detection task marked by the non-spatial feature repetition benefit increased with increasing percentiles, suggesting that retrieval has a stronger impact on later responses, this pattern was fully absent in the visual detection task. In the latter, faster and slower responses were unaffected by non-spatial feature repetition benefits. Additionally, we found that IOR became stronger with increasing response times, a pattern that was significant for visual targets, and approached significance for auditory targets. This suggests that IOR takes time to emerge (see also, e.g., Chao et al., 2020; Panis & Schmidt, 2020; Taylor & Ivanoff, 2005). Hence, the non-occurrence of binding effects in visual detection and localization performance is caused by task demands and the modality involved.

In the auditory detection task, repeating or changing the location and non-spatial features had two separate effects on task performance: A pattern congruent with IOR and a pattern congruent with S-R binding. There have been some observations of IOR being affected by systematically repeating or changing a stimulus’ non-spatial feature, leading to what is often referred to as “non-spatial IOR”. Interestingly, in these tasks (typically cue-target designs, in which a response is only given to the second of two targets in a sequence; for a target-target design, see Chao et al., 2020), IOR is larger if the non-spatial feature of the target repeats (e.g., Fox & De Fockert, 2001; Law et al., 1995), specifically at location repetitions (Hu et al., 2011, 2013). Such non-spatial IOR effects have been observed for auditory stimuli as well, that is, a benefit for a frequency change (e.g., cue-target condition in Experiment 1 of Mondor & Leboe, 2008), referred to as frequency-based IOR (e.g., Mondor et al., 1998; Prime & Ward, 2002), also at location repetitions (Chen et al., 2007). In other words: *Changing* the non-spatial feature while repeating its location accelerates responding. In contrast, Mondor and Leboe (2008; consecutive responding condition in Exp. 1; 300 ms RSI in Exp. 3) and the current study showed that *repeating* the non-spatial feature accelerates responding contrary to changing it. Congruent with that, Prime and Ward (2002) did not observe frequency-based IOR, but rather frequency-based facilitation in a target-target design, that is, when participants responded to both cue and target (see also Spence & Driver, 1998, for how responding to auditory and visual targets in a cue-target design is differently affected by the previous cue-target trial). The interpretation can be two-fold: On the one hand, it could be argued that the integration of motor components into an event file during the prime display is the crucial component to observe binding effects in auditory detection performance revealed by a benefit for frequency repetition – which can blur frequency-based IOR effects in cue-target designs (see Chen et al., 2007; Mondor et al., 1998; cue-target condition of Experiment 1 in Mondor & Leboe, 2008). On the other hand, it could be argued, that in a cue-target design, the “no-response” to the cue followed by the detection response to the target is processed as a response change – for which a feature change is beneficial (e.g., Frings et al., 2020).

Mondor and Leboe (2008) discuss the difference in data patterns in their first experiment also in the context of retrieval of event files (Hommel, 2004), that is, retrieval of non-spatial information modulated by response repetitions/non-repetitions. This explanation still holds up: When participants in Mondor and Leboe (2008) gave a detection response to both targets (Exp. 1: Consecutive condition), a benefit for frequency repetition emerged (i.e., a benefit for response repetition with non-spatial feature repetition, and interference for response repetition with non-spatial feature change). In contrast, when participants gave no response to the first target, but a detection response to the second target (Experiment 1: Cue-target condition), a benefit for frequency change emerged (i.e., a benefit for response change with non-spatial feature change, and interference for response change with non-spatial feature repetition). Although data collection for said conditions was between-subjects, the emerging data pattern could still be interpreted as depicting partial repetition costs (Frings et al., 2020; Hommel, 1998, 2004). Moreover, the different observations for cue-target vs. target-target/prime-probe designs suggest that it is not a general benefit for pitch repetition (e.g., in the sense of priming of a certain feature) per se, but rather the interplay of non-spatial feature repetition and change with response repetition and change. This explanation has potentially far-reaching theoretical implications: What is sometimes referred to as frequency-based IOR in cue-target designs could also be interpreted as a benefit of response change and feature change over response change and feature repetition – the latter causing partial repetition costs (Hommel, 2004).

Finally, we interpreted the current data pattern as the occurrence or absence of S-R binding effects in action control paradigms due to target modality. However, other theories or effects might have (additively) influenced the data pattern or are congruent with it. The benefit of a non-spatial feature repetition could be interpreted as the cognitive system assigning more “weight” to detect a repeating frequency in the sense of *dimensional weighting* (Found & Müller, 1996; Müller et al., 1995; Müller & Krummenacher, 2006; however, note that in *visual* search, dimensional weighting emerges primarily if a dimension – like color, orientation, etc. – repeats, whereas a repetition or change of the specific dimension feature – the specific color, the specific orientation – sometimes plays a minor role; see Found & Müller, 1996) or priming of certain features (e.g., as in some visual search tasks; Becker & Horstmann, 2009; Kruijne & Meeter, 2016). According to the bypass rule (e.g., Fletcher & Rabbitt, 1978; Krueger & Shapiro, 1981; see Mondor & Leboe, 2008, for a discussion in the context of auditory detection performance) and stimulus-response repetition heuristics (e.g., Pashler & Baylis, 1991), participants have the tendency to repeat the response if stimulus information repeats. As in our study participants could only repeat the response, a non-spatial feature change might have caused interference (however, see Frings et al., 2007, for a discussion of the bypass rule in the context of retrieval). However, note that these explanations would only explain the frequency repetition benefit in the auditory detection task – and neither the absent effect for visual targets nor the occurrence of IOR in both tasks. In other words, we still observed modality differences (see, e.g., Quinlan & Hill, 1999, for the bypass rule in the context of visual versus auditory targets). Thus, the current results are not only congruent with assumptions in multiple frameworks, but also offer the possibility to investigate modality dependencies in such.

The present study is the first showing that binding processes of the exact same action – signaling the detection of a stimulus – are affected by stimulus modality. Thus, our data show a limitation for binding approaches in action control theories (e.g., Frings et al., 2020) which do so far not propose that binding processes depend on target modality. Further, because the results fit well with previous observations of modality differences in paradigms discussed in the context of binding in action control (e.g, spatial negative priming; Mayr et al., 2009; Möller et al., 2016), they might also be of interest in other paradigms investigating different modalities or the combination of such, like in task switching (e.g., Stephan & Koch, 2011; Strobach et al., 2012) or action planning (e.g., Keller & Koch, 2006). By that, it can be deduced if modality differences arise, for example, in more complex paradigms, and, ultimately, if modality differences generalize to other fields in action control.

## Conclusion

Detecting a visual or auditory target is the exact same action in terms of S-R binding approaches; yet, we could show that only auditory, but not visual detection performance is influenced by feature repetition benefits that are assumed to underlie binding effects. Thus, we observed another possible *boundary of binding*: In detection tasks, binding effects are modality-dependent. On a somewhat larger note, our results question the ubiquity of S-R binding in action control and might help to define the circumstances for binding and retrieval affecting performance.

## Appendix

**Table 1:** Averaged percentiles in ms of probe responses separate for conditions and target type

Condition	Targets	Percentiles				
		10th	25th	50th	75th	90th
FRLR	Auditory	198	235	273	319	362
	Visual	165	202	238	276	312
FRLC	Auditory	198	236	272	313	358
	Visual	163	198	232	266	302
FCLR	Auditory	199	239	281	332	381
	Visual	164	203	237	274	317
FCLC	Auditory	198	236	275	321	374
	Visual	166	198	231	267	300

## Footnotes

1 Mondor and Leboe (2008) also investigated a cue-target design (Experiment 1: Cue-target condition), as well as observed IOR in dependence of adjusting the RSIs between prime and probe, as well as probe and prime (Experiment 2 and 3). However, here we are only interested in the conditions that showed a benefit for frequency repetition. The pattern resulting from a cue-target design will be compared to the target-target/prime-probe design in the General Discussion.

2 Due to this, catch trials were primarily catch trials for prime responses (see also footnote 5 in Schöpper, Hilchey, et al., 2020). However, we adopted this component from Schöpper, Hilchey, et al. (2020) to have a design that is identical to the original study, so that any changes in data outcome could be ascribed to the stimuli and/or target modality used.

3 Some studies found slower responding in trials following catch trials (e.g., Alegria, 1978; Jongen & Smulders, 2007), and one might muse if effects of non-responding on a preceding trial might have affected subsequent responses in our study (e.g., in the sense of Go/No-Go tasks, Burnham, 2013). Accordingly, we ran an additional analysis by separating those trials following catch trials from those following „common“ trials, and added preceding trial type (catch vs. common) as a factor to the 2 x 2 ANOVA reported above. Participants were slower following catch trials (293 ms) compared to common trials (276 ms), *F*(1, 39) = 21.74, *p* < .001, ${\upeta}_p^2$ = .36. Interestingly, preceding trial type also modulated the relevant effect of non-spatial feature relation, *F*(1, 39) = 7.72, *p* = .008, ${\upeta}_p^2$ = .17, in that the benefit of frequency repetition (287 ms) over change (300 ms) was larger for trials following catch trials than those following common trials (FR: 274 ms; FC: 279 ms). It could be argued that overall slower responding due to preceding catch trials allowed more time for retrieval to unfold (see also General Discussion). Preceding trial type did not modulate the remaining effects (both *F* ≤ 0.04).

4 One might argue that presenting the faces on the left and right would be more comparable to the auditory detection task. However, we wanted to keep the visual detection task in the current study as close as possible to the visual detection task in Schöpper, Hilchey, et al. (2020). Authors have used targets appearing above, below, left, or right to the fixation cross to investigate IOR (e.g., Kwak & Egeth, 1992). While positions in the visual field can have an effect on reaction times in detection tasks (e.g., Spalek & Hammad, 2004), their impact on IOR is debated (Snyder & Schmidt, 2014; however, see Soballa et al., in prep.).

5 As with Experiment 1, participants were slower following catch trials (244 ms) compared to common trials (234 ms), *F*(1, 39) = 10.64, *p* = .002, ${\upeta}_p^2$ = .21. Neither did preceding trial type significantly modulate non-spatial feature relation, *F*(1, 39) = 2.28, *p* = .139, ${\upeta}_p^2$ = .06 (Catch trials: FR: 246 ms; FC: 241 ms; Common trials: FR: 233 ms; FC: 234 ms), nor did it modulate the remaining effects (both *F* ≤ 0.02).

6 Participants barely made errors in both tasks; we therefore did not analyze error rates in a manner comparable to reaction times. Overall error rates (i.e., a missed probe response after a given prime response) were 0.99 % in the auditory detection task and 1.10 % in the visual detection task, and did not differ when compared with an independent sample t-test (two-sided), *t*(78) = 0.34, *p* = .738, *d* = 0.08.
